# Predator-induced changes of female mating preferences: innate and experiential effects

**DOI:** 10.1186/1471-2148-11-190

**Published:** 2011-07-04

**Authors:** David Bierbach, Matthias Schulte, Nina Herrmann, Michael Tobler, Stefan Stadler, Christian T Jung, Benjamin Kunkel, Rüdiger Riesch, Sebastian Klaus, Madlen Ziege, Jeane Rimber Indy, Lenin Arias-Rodriguez, Martin Plath

**Affiliations:** 1 Department of Ecology & Evolution, J.W. Goethe University Frankfurt, Siesmayerstrasse 70-72, D-60054 Frankfurt am Main, Germany; 2 Institute of Biochemistry & Biology, Unit of Animal Ecology, University of Potsdam, Maulbeerallee 1, 14469 Potsdam, Germany; 3 Department of Zoology, Oklahoma State University, 501 Life Sciences West, Stillwater, OK 74078, USA; 4 Department of Biology & W. M. Keck Center for Behavioral Biology, North Carolina State University, 127 David Clark Labs, Raleigh, NC 27695-7617, USA; 5 Department of Biological Sciences, National University of Singapore, 14 Science Drive 4, Singapore 117543; 6 División Académica de Ciencias Biológicas, Universidad Juárez Autónoma de Tabasco (UJAT), C.P. 86150 Villahermosa, Tabasco, México

**Keywords:** Sexual selection, female choice, non-independent mate choice, predator recognition, *Poecilia mexicana*

## Abstract

**Background:**

In many species males face a higher predation risk than females because males display elaborate traits that evolved under sexual selection, which may attract not only females but also predators. Females are, therefore, predicted to avoid such conspicuous males under predation risk. The present study was designed to investigate predator-induced changes of female mating preferences in Atlantic mollies (*Poecilia mexicana*). Males of this species show a pronounced polymorphism in body size and coloration, and females prefer large, colorful males in the absence of predators.

**Results:**

In dichotomous choice tests predator-naïve (lab-reared) females altered their initial preference for larger males in the presence of the cichlid *Cichlasoma salvini*, a natural predator of *P. mexicana*, and preferred small males instead. This effect was considerably weaker when females were confronted visually with the non-piscivorous cichlid *Vieja bifasciata *or the introduced non-piscivorous Nile tilapia (*Oreochromis niloticus*). In contrast, predator experienced (wild-caught) females did not respond to the same extent to the presence of a predator, most likely due to a learned ability to evaluate their predators' motivation to prey.

**Conclusions:**

Our study highlights that (*a*) predatory fish can have a profound influence on the expression of mating preferences of their prey (thus potentially affecting the strength of sexual selection), and females may alter their mate choice behavior strategically to reduce their own exposure to predators. (*b*) Prey species can evolve visual predator recognition mechanisms and alter their mate choice only when a natural predator is present. (*c*) Finally, experiential effects can play an important role, and prey species may learn to evaluate the motivational state of their predators.

## Background

Female mate choice has long been recognized as a major driver for character displacement [1-3], or the evolution of novel male traits [4,5], but can also play a vital role during speciation processes by promoting reproductive isolation through assortative mating [6,7]. Many mating preferences are innate [1]; still, various extrinsic factors (ecological constraints) may affect individual mating decisions [8-10], such as altered possibilities for mate quality assessment due to increased costs of mate searching [11,12]. Additionally, the social environment of the choosing individual is known to affect the strength [13-17] or even the direction of mating preferences [18-20].

Another decisive factor acting upon the expression of (female) mating preferences in natural systems is predation risk [21-26]. Female sand gobies (*Pomatoschistus minutus*), for example, normally prefer larger and more colorful males, but were found to be less choosy when exposed to a predator [27]. Decreased choosiness due to the presence of predators was also reported for male mate choice in the sex-role reversed pipefish *Syngnathus typhle *[28]. Furthermore, studies on guppies (*Poecilia reticulata*) revealed that females when facing a predator switch towards associating with less colorful males [29,30], and females of the green swordtail (*Xiphophorus hellerii*) that usually prefer males with long swords, switch their preference towards males with short swords when exposed to videos showing successful predator attacks [31].

Such behavioral alterations can be interpreted as a tactic employed by the choosing individuals to reduce their own exposure to predators, as brightly colored males attract predators to the area, and by having more predators in the area females' predation risk is increased [32,33]. For example, brightly colored males in the Trinidadian guppy are more vulnerable to predation by the predatory cichlids *Aequidens pulcher *and *Crenicichla alta *than drabber ones [34-40] and females that preferentially associate with such brightly colored males will obviously face an equally high predation risk.

Our present study was designed to investigate predator-induced changes of female mating preferences in the Atlantic molly (*Poecilia mexicana*, Poeciliidae). *Poecilia mexicana *males show a pronounced polymorphism in body size and coloration [41-43], and females prefer larger, more colorful, dominant males as mating partners [42,44]. At the same time, large molly males are more conspicuous to predators, as exemplified by studies of avian predation on the related sailfin molly, *P. latipinna *[45], or predation by giant water bugs on *P. mexicana *[46,47]. Hence, female mollies might increase their own risk of being attacked by a predator when associating with larger males.

The present study involved piscine predators of *P. mexicana*, and used cichlid species that regularly co-occur with Atlantic mollies in the same habitats [48-50]. Even though prey choice experiments on the cichlid species considered here (especially '*Cichlasoma' salvini*) still need to be conducted, it seems straightforward to assume a preference for larger prey, as another cichlid, the aforementioned pike cichlid (*Crenicichla alta*), also prefers larger guppies as prey [51,52]. Adult *C. salvini *and female *P. mexicana *show roughly comparable relationships in body size as do *C. alta *and female *P. reticulata *(ratio SL *P. reticulata*, 15-28 mm [53] v. maximum SL *C. alta*, about 160 mm [53,54]: 0.10-0.18; ratio SL *P. mexicana*, 35-40 mm [[55], this study] v. maximum SL *C. salvini*, about 220 mm [54]: 0.16-0.18), so it seems reasonable to argue that *C. salvini *would not be gape-limited when preying on average-sized *P. mexicana *females. Based on these considerations, we predicted that *P. mexicana *females should alter their mate choice behavior when facing predation risk by piscivorous cichlids; specifically, females should associate with small rather than large males when a piscine predator is around to minimize their own risk of being attacked.

Simultaneously, we asked whether females are able to recognize piscivorous predators and distinguish them from similar non-piscivorous species on the basis of visual cues. We hypothesized that only piscivorous predators would lead to a reversal of female preferences. To test our predictions, we conducted dichotomous female mate choice tests (association preference tests) and repeated the tests while either a natural molly predator ('*Cichlasoma' salvini*, Cichlidae) or a non-piscivorous fish (three types: two cichlids and another poeciliid, the green swordtail, *Xiphophorus hellerii*) were presented. This design allowed us to compare changes in the expression of female mating preferences from the 1^st ^to the 2^nd ^part of the tests among four different contexts (*i.e*. predator treatments). At least for the treatment involving green swordtails-a mainly detritivorous fish [56] that often occurs in the same microhabitats as *P. mexicana *in southern México and is of similar body size [48,49]-we predicted that *P. mexicana *females should not alter their preferences. This treatment, therefore, served as a control to test whether females would be consistent in their mate choice behavior, or if any changes occurred over the course of the experiment that would not be attributable to the presence of a predator.

Finally, we asked the interrelated question of whether visual predator recognition (and the correlated specific responses of females to piscivorous as opposed to non-piscivorous fishes) is innate, or whether also experiential effects/learned predator avoidance could play a role. Comparing the responses of lab-reared (predator-naïve) and wild-caught (predator-experienced) females allowed us to disentangle innate and learned components of predator recognition [57,58]. We hypothesized that (*a*) if visual predator recognition mechanisms are entirely innate, then both lab-reared and wild-caught females should respond to the presence of a molly predator during their mate choice, while (*b*) if experiential effects are important, then a response might not be observable in one of the two different female groups (predator-naïve or wild caught).

In summary, our study aims to answer the following questions: (1) Do *P. mexicana *females change their mating preferences when exposed to a visually presented piscine predator? (2) Are molly females able to distinguish between predatory species and similar-shaped non-predatory ones on the basis of visual cues alone? (3) Does predator experience affect females' responses to a predator during mate choice?

## Methods

### Origin and maintenance of study animals

Predator-naïve (lab-reared) test fish were first generation descendents of fish collected from the Río Oxolotán near the town of Tapijulapa, Tabasco, México. They were reared in large (6,000 liters) fish culture tanks at the aquaculture facilities of the Academic Division for Biological Sciences at Universidad Juárez Autónoma de Tabasco (DACBIOL-UJAT) in Villahermosa. Prior to the mate choice experiments fish were kept separated by sex in well-aerated 70-liter tanks at a temperature of 27°C under a natural, approximately 12: 12 hours light: dark cycle.

Predator-experienced (wild-caught) fish were collected in the Río Ixtapangajoya near the city of Teapa, which just like the Río Oxolotán is a tributary of the Río Grijalva. Streams in the Río Grijalva system are widely interconnected in the lowlands at least during the wet season [59], and both collection sites at the Río Oxolotán and the Río Ixtapangajoya are within 20 km river distance. Furthermore, extensive haplotype sharing (based on mt-DNA markers) between mollies from both rivers was recently uncovered [60], so it was legitimate to treat fish from both sampling sites as representatives of the same population for the purpose of this study. Upon capture, fish were transferred into closed and aerated 38 L (43 × 31 × 32 cm) black Sterilite^® ^containers and brought immediately to the laboratory at DACBIOL-UJAT, where they were kept separated by sex in aerated 70-liter tanks for 24 hours to allow acclimation to lab conditions. In the laboratory all fish were fed once a day *ad libitum *with commercially available flake food.

There is no direct way of easily assessing reproductive status in wild-caught females. However, due to their size (mean SL 34.5 ± 0.6 SEM), all females used in this study were most likely in their reproductive stage (see [55]). Certainly, they may have varied in their reproductive status, but judging from their abdominal distention most of them were likely pregnant. However, as poeciliid females are more receptive to male approaches for few days after giving birth [61,62], we avoided using post partum females for our tests.

### Mate choice tests

**Figure 1:**
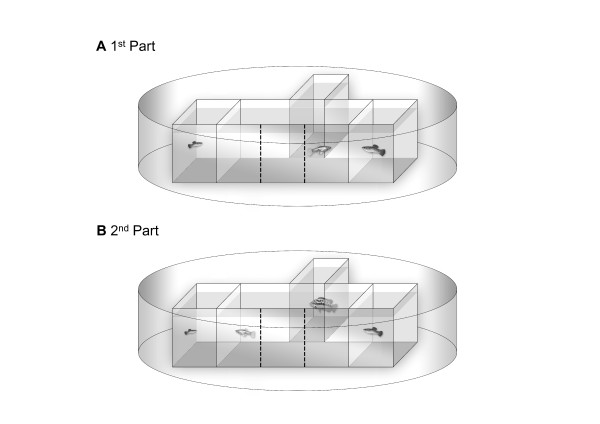
**Schematic view of the experimental set-up used to determine female preferences under predation risk**. **A**. During the 1^st ^part of the tests the focal female could choose to associate with either of two stimulus males (large and small). **B**. Tests were repeated while a predator (here: *C. salvini*) was presented in an adjacent tank (2^nd ^part). Fishes are not drawn to scale.

Mate choice experiments were conducted as part of the University course 'Tropical Ecology' of the University of Frankfurt between September 11^th ^and October 17^th ^2010 at DACBIOL-UJAT, Villahermosa, Tabasco, México. Tests were conducted in three identical portable test tanks (42.6 × 30 × 16.5 cm) built from UV-transparent Plexiglas. Each tank was visually divided into three equally-sized zones by black marks on the outside. The central zone was designated the neutral zone, the two lateral zones as preference zones. Two stimulus males were presented in two smaller auxiliary tanks (19.5 × 30 × 14.5 cm) on either side of the test tank. Hence, the focal female could choose a mate on the basis of visual cues. To avoid disturbance from the outside, we set up all test tanks in large oval tubs that were filled with water to the level inside the test tanks (Figure 1). The entire set-up was placed on a shelf of about 1 m height, and the observer was standing approximately 1.5 to 2 m away from the test apparatus and observed the fish from diagonally above. Therefore, the test fish maximally saw the observer's head, which further helped minimize disturbance of the test fish.

Molly males constantly attempt to mate with females (e.g., even directly upon capture [63]; D.B., R.R. and M.P. personal observation). Hence, it is likely that time spent by a female in association with a given male (leading to physical proximity) facilitates male copulation attempts by that particular male. Furthermore, a recent study by Walling et al. [64] experimentally demonstrated that female association preferences actually do translate into male reproductive success in green swordtails, *Xiphophorus hellerii *(Poeciliidae). We are therefore confident that association preferences in fact translate into more copulations with the preferred male also in *P. mexicana*.

Before each trial, a large and a small stimulus male was placed into either auxiliary tank [mean (± SEM) SL; large: 45.2 ± 0.5 mm; small: 32.8 ± 0.5 mm]. Then, a female was introduced into the test tank (SL 34.5 ± 0.6 mm). The choice tanks used in this study were relatively small, so focal females were able to see both stimuli at all times. Test fish would typically freeze on the bottom of the test tank for few seconds (to some minutes) after they were introduced, so a trial began only after the focal female had started to swim freely in the water column. We measured the time the female spent in each preference zone during a 5-min observation period. To detect side biases, the stimuli were switched between sides immediately after the first 5-min observation period and measurement was repeated. This episode is henceforth called the 1^st ^part of the tests.

**Figure 2:**
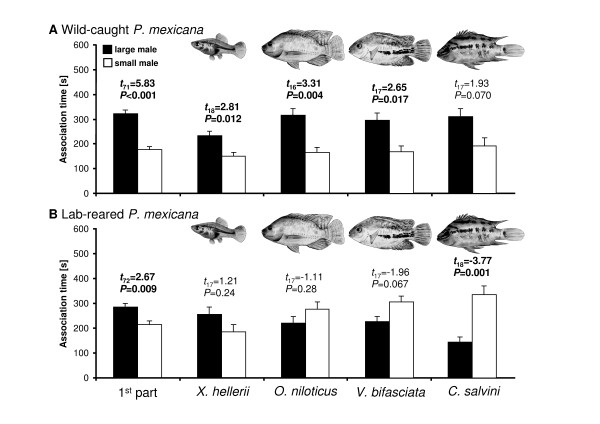
**Time females spent in association with the large and the small stimulus males during the 1^st ^part of the choice tests (*left*) and during the 2^nd ^part of all four predator treatments [treatments (1)-(4), from *left *to *right*]**. Depicted are association times (± SEM). Test results are from paired *t*-tests.

**Figure 3:**
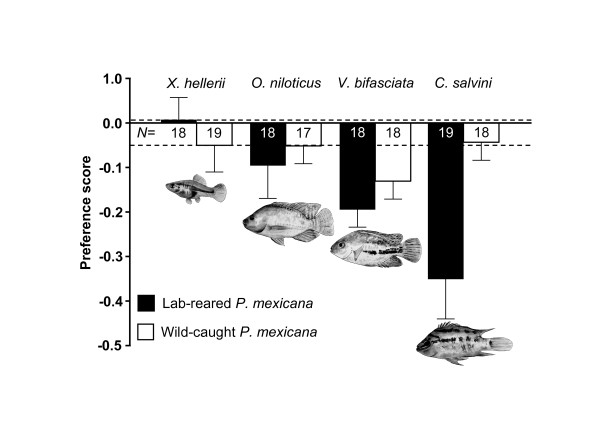
**Changes in Atlantic molly females' individual mating preferences when confronted with a piscine predator**. Depicted are preference scores (see main text), whereby negative values indicate that female preferences for the initially preferred male decreased in strength.

During the 2^nd ^part of our choice tests, we presented a heterospecific audience in a transparent box (a plastic mouse cage measuring 23 × 15 × 16.5 cm) next to the neutral zone outside of the main test tank (Figure 1). The audience was one of four different fish species (*treatments 1-4*), three of which are found in sympatry in natural *P. mexicana *habitats in the Río Grijalva drainage [48-50]. The four species differed in nutritional ecology (piscivorous v. non-piscivorous) as well as body shape and coloration (see inserted drawings in Figures 2 and 3). Females were assigned randomly to each of the four treatments.

In treatment (1), females were confronted with a green swordtail (*X. hellerii*) female (SL: 38.9 ± 1.4 mm) which served as a control for consistency of female preferences, since *X. hellerii *is a related species with a similar size, appearance, and ecology. In treatment (2) we presented focal females with a Nile tilapia (*Oreochromis niloticus*; 71.0 ± 1.0 mm). This species was introduced to Mexican freshwaters during the second half of the 20^th ^century [65,66], and the related species *O. aureus *is found sympatric with *P. mexicana *[48]. Nile tilapia is described as a phytoplanktivorous filter feeder [67] and also ingests various plants, detritus, and insects like chironomid larvae [68]. In treatment (3), we confronted females with the algi- and detrivorous cichlid *Vieja bifasciata *(91.9 ± 2.8 mm; [69,70]), which is also common in the natural habitats of *P. mexicana *[48]. Finally, in treatment (4) we used '*Cichlasoma' salvini *(98.2 ± 4.0 mm), which is a native omnivorous cichlid in southern México [70,54] and also includes mollies in its diet (M.P. personal observation). We chose this cichlid species because it is common at both sampling sites ([48]; authors, personal observation) while other, more specialized piscivorous predators (such as *Centropomus undecimalis *[48]) are comparatively rare and, due to their large body size, less suited for laboratory experiments. We repeated measurements of female association times (including switching of side-assignments of the two stimulus males) as described for the 1^st ^part of the experiments.

### Statistical analysis

All data were tested for normality using Kolmogorov-Smirnov-tests. Data are generally given as mean ± standard error (SEM). To evaluate female preferences for large male body size we compared the amount of time focal females spent near the large and small stimulus males during the initial preference test (1^st ^part) as well as during the 2^nd ^part of all four treatments using paired *t-*tests.

Our central question was whether females would alter their individual choice decisions under the influence of a heterospecific audience. We, therefore, calculated a score expressing the change of female mating decisions [13] as the difference between individual females' relative association times near the initially preferred male during the 2^nd ^part (with predator present) and relative association times near the same male during the 1^st ^part (without predator), such that no change in female preferences would lead to a score of zero, negative values would indicate that the focal females spent less time near the initially preferred male in the 2^nd ^part of a trial, and positive values would indicate that females spent relatively more time near the initially preferred male. Scores were compared among treatments using a fully factorial univariate General Linear Model (GLM) with 'treatment' and 'predator experience' as independent variables. We included 'focal female body size', 'audience body size' as well as 'stimulus male body size difference' (SL large stimulus male-SL small stimulus male) as covariates in our first analysis, but removed 'stimulus male body size difference' (*F*_1,144 _= 0.16, *P *= 0.69) and 'audience body size' (*F*_1,135 _= 1.74, *P *= 0.19) from our final model as they had no statistically significant effects. Where covariates were significant in our final model, standardized residuals from GLM were used to calculate *post hoc *Pearson correlations.

## Results

### Female preference for large male body size

Focal females, both from the lab and from the wild, spent significantly more time in association with larger males during the 1^st ^part of the preference tests (Figure 2). Wild-caught females retained a significant preference for the larger of the two stimulus males during the 2^nd ^part in treatment (1) (*X. hellerii*), treatment (2) (*O. niloticus*), and treatment (3) (*V. bifasciata*). Females also spent more time with the larger male in treatment (4) (*C. salvini*), but this effect was not statistically significant (*P *< 0.1; Figure 2B).

During the 2^nd ^part of the tests lab-reared (*i.e*. predator-naïve) females tended to spend more time in association with the larger male in treatment (1), while females tended to spend more time with the smaller male in treatments (2) and (3), but in neither case was the difference statistically significant (Figure 2A). Notably, females spent significantly more time near the smaller male in treatment (4) (with *C. salvini*).

### Changes in individual female preferences

The degree to which individual female preferences changed from the 1^st ^to 2^nd ^part of the tests (preference score; Figure 3) differed significantly between the two types of females (*i.e*. lab-reared and wild-caught fish; Table 1) as well as among audience treatments (see effect of the factor 'treatment' in Table 1). However, as indicated by a significant interaction term of 'treatment by predator experience' (Table 1), females of the two groups responded differently to the four different types of audience. The pattern is revealed in Figure 3: predator-naïve, lab-reared females altered their preferences when *C. salvini *was presented, while wild-caught females did not show such responses.

**Table 1:** Results of a full-factorial univariate GLM using preference scores (see main text) as dependent variable.

Effect	*df*	Mean square	*F*	*P*
Treatment	3	0.246	3.732	0.013
Predator experience	1	0.241	5.074	0.026
Focal female body size	1	0.175	4.884	0.029
Treatment × predator experience	3	0.210	5.680	0.001
Error	136	0.313		

**Figure 4:**
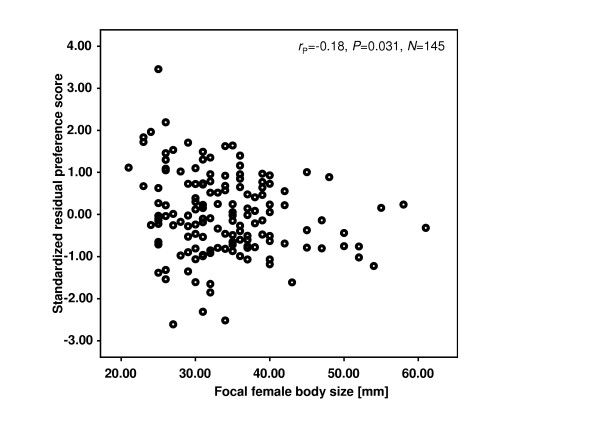
**The correlation between the strength of change of female preference (preference score) and female body size (SL)**. Standardized residuals were obtained from a General Linear Model (see Table 1). Test results are from Pearson correlation. Note that larger females were more likely to change their preferences over the course of the experiment.

In the GLM 'focal female body size', too, had a significant effect (Table 1), and a *post hoc *Pearson correlation using standardized residuals revealed a significant negative correlation (*r*_P _= -0.18, *P *= 0.031, *n *= 145; Figure 4); in other words: larger females were generally less consistent in their mate choice.

## Discussion

Predation plays a central role as a natural selection factor [71] and affects various aspects of prey species' ecology and behavior. In many cases the male sex is more susceptible to predation [21,72-76]. For instance, several studies on Trinidadian guppies (*Poecilia reticulata*) suggest that predation risk has a profound impact on the kind of mating tactics employed by males as well as the evolution of color patterns [62,77,78]. Males from 'high-predation' streams, which are characterized by the presence of large cichlid predators, show less courtship behavior and more sneaky copulations, while evolving less conspicuous coloration [62,79-81].

In the present study we asked (*a*) if female Atlantic mollies (*Poecilia mexicana*) would adjust their mating preferences strategically to the presence of a predator, (*b*) if females would distinguish between piscivorous and similar-shaped non-piscivorous fishes on the basis of visual cues alone by responding alternatively, and (*c*) if prior experience with predators affects females' responses. We found predator naïve (lab-reared) females to spend more time near the initially non-preferred (smaller) male when confronted with a cichlid during the 2^nd ^part of the tests, but the strongest response-with females exhibiting a significant preference for smaller stimulus males-was found in treatment (4), which involved *C. salvini*, an omnivorous species that is known to also prey on mollies. A similar effect was reported for green swordtail (*Xiphophorus hellerii*) females [31]. Females of that species showed a predilection for males with an elongated caudal fin (a sword-like structure) when no predator was around, but preferred males sporting short swords when a predatory cichlid (*Petenia splendida*) was presented. Since another visual, diurnal swordtail predator, the characid *Astyanax mexicanus*, has been shown to prefer males with long swords [33], females are thought to reduce their own predation risk by associating with small-sworded males under predation threat.

Females in our study did not change their preferences in treatment (1), which involved a swordtail female as audience. This result is congruent with a previous study reporting on highly consistent mating preferences of *P. mexicana *females, irrespective of whether choice tests were conducted in front of a con- or heterospecific poeciliid female, or without an audience [82]. By contrast, male poeciliids strongly respond to the presentation of a conspecific audience male by reducing their sexual activity and preference expression [83,84]-a response that has been interpreted as a tactic employed by males to prevent rivals from copying their mate choice [83,85,86]. Our treatment (1), therefore, served as a baseline to make sure that focal females in our present study would indeed be consistent in mate choice over the course of the experiment when no predator was present.

Larger females were generally less consistent in their mate choice than smaller ones (see Figure 4). We do not have a compelling explanation for this surprising finding at hand. However, we tentatively argue that as large *P. mexicana *females are preferred by males [13,14,87] and thus, will attract more males in natural populations (Bierbach et al., unpublished data), larger females could simply be less inclined to associate with a high quality mate for an extended period of time since they are more likely to be approached by yet another (large) male in the near future.

### Innate and experiential effects

Our finding that at least lab-reared (predator-naïve) females responded differentially to the four types of audience points towards an innate component of visual predator recognition [88]. Surprisingly, wild-caught (predator experienced) females in our study did not respond to the same extent to a predator as did naïve females. Indeed, wild-caught females still tended to express a preference for large male body size during the 2^nd ^part of the tests even when the predatory *C. salvini *was presented. At first sight these results seem to contradict other studies on guppies reporting on stronger anti-predator responses in predator experienced (wild-caught) fish compared to lab-reared fish from the same population [89,90].

**Figure 5:**
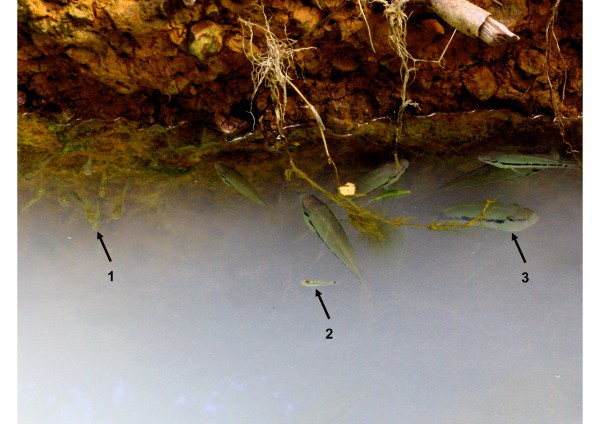
**A shoal of adult Atlantic mollies, *P. mexicana *(*left arrow, 1*), as well as another poeciliid (*Heterandria bimaculata; middle, 2*) in close proximity to a group of *C. salvini *(*right, 3*)**. Note that the mollies showed no obvious fright response. The photo was taken at a site in a clear-water affluent to the "El Azufre", a sulfidic stream in the Cueva del Azufre system [48]. Traces of calcium sulphate give the water a milky appearance.

So, why did field-collected females (*i.e*. females that had experienced predators in their natural habitat) not respond to the visual presence of *C. salvini*, while lab-reared females did so? First, for our experiments we had to use subadult predator specimens that were certainly smaller than the size classes typically preying on mollies in natural habitats. Secondly, even though *C. salvini *is common in natural molly habitats in southern México [48,70], sympatric *P. mexicana *may not always be under immanent predation threat [91]: For example, predators will not represent any risk for some time after a successful catch. Accordingly, we regularly observe groups of *P. mexicana *(and other poeciliids) in close proximity to groups of *C. salvini *in their natural habitat, where mollies typically would not show any obvious fright responses even towards large, adult predators (Figure 5). Similar observations were made in the predator-prey interaction between pike cichlids (*Crenicichla *spp.) and Trinidadian guppies (*P. reticulata*) [92]. We, therefore, propose that females in nature learn to evaluate the actual level of threat posed by surrounding piscine predators. Experiential effects on anti-predator behaviors are well known from fishes [58,88], and context-dependent anti-predator responses may be adaptive as they allow saving energy and time that would be wasted when prey species respond indiscriminately [62]. Guppies, for example, display stronger anti-predator responses toward a hungry predator, and predator-experienced fish from a high-predation locality respond more strongly to a hungry predator than those from a low predation locality [91]. We suggest that wild-caught *P. mexicana *females in our present study have learned to evaluate the predators' motivational state. Predators in our experiment were certainly somewhat stressed due to handling and the relatively small dimensions of their experimental compartment and, therefore, could have presented focal females with visual cues regarding their (lack of) motivation for imminent predation. Future studies will have to answer the question of how exactly mollies determine predators' motivation, but changes in color and movement patterns are the most likely candidates [88].

## Conclusion

Overall, our present study highlights that (*a*) predatory fish can influence the expression of mating preferences of their prey, and females may alter their mate choice behavior strategically to reduce their own exposure to predators. (*b*) Prey species can evolve visual predator recognition mechanisms and alter their mate choice according to the identity of the audience. (*c*) Finally, experiential effects also play a role, and prey species may learn to evaluate the motivational state of their predators. Altogether then, our present study underscores the important role played by environmental factors on the expression of mating preferences and adds to our understanding of the multiple pathways by which predation affects prey populations.

## Competing interests

The authors declare that they have no competing interests.

## Authors' contributions

All authors collected fish and jointly performed the experiments at DACBIOL-UJAT. DB, RR, MT, NH, MS and MP wrote the first draft of the manuscript; drawings are by MZ. All authors read and approved the final manuscript.
